# The predictive value of TNF family for pulmonary tuberculosis: a pooled causal effect analysis of multiple datasets

**DOI:** 10.3389/fimmu.2024.1398403

**Published:** 2024-05-21

**Authors:** Wenxiu Mo, Zhezhe Cui, Jingming Zhao, Xiaomin Xian, Minying Huang, Jun Liu

**Affiliations:** ^1^ School of Public Health and Management, Youjiang Medical University for Nationalities, Baise, China; ^2^ Guangxi Key Laboratory of Major Infectious Disease Prevention and Control and Biosafety Emergency Response, Guangxi Key Discipline Platform of Tuberculosis Control, Guangxi Centre for Disease Control and Prevention, Nanning, China; ^3^ School of Public Health, the Key Laboratory of Environmental Pollution Monitoring and Disease Control, Ministry of Education, Guizhou Medical University, Guiyang, China; ^4^ Department of Neurosurgery, Liuzhou People’s Hospital, Liuzhou, China

**Keywords:** pulmonary tuberculosis, tumor necrosis factor, Mendelian randomization, colocalization, pooled causal effects

## Abstract

**Objective:**

Despite extensive research on the relationship between pulmonary tuberculosis (PTB) and inflammatory factors, more robust causal evidence has yet to emerge. Therefore, this study aims to screen for inflammatory proteins that may contribute to the susceptibility to PTB in different populations and to explain the diversity of inflammatory and immune mechanisms of PTB in different ethnicity.

**Methods:**

The inverse variance weighted (IVW) model of a two-sample Mendelian Randomization (MR) study was employed to conduct causal analysis on data from a genome-wide association study (GWAS). This cohort consisting PTB GWAS datasets from two European and two East Asian populations, as well as 91 human inflammatory proteins collected from 14,824 participants. Colocalization analysis aimed to determine whether the input inflammatory protein and PTB shared the same causal single nucleotide polymorphisms (SNPs) variation within the fixed region, thereby enhancing the robustness of the MR Analysis. Meta-analyses were utilized to evaluate the combined causal effects among different datasets.

**Results:**

In this study, we observed a significant negative correlation between tumor necrosis factor-beta levels (The alternative we employ is Lymphotoxin-alpha, commonly referred to as LT) (*P* < 0.05) and tumor necrosis factor receptor superfamily member 9 levels (TNFRSF9) (*P* < 0.05). These two inflammatory proteins were crucial protective factors against PTB. Additionally, there was a significant positive correlation found between interleukin-20 receptor subunit alpha levels (IL20Ra) (*P* < 0.05), which may elevate the risk of PTB. Colocalization analysis revealed that there was no overlap in the causal variation between LT and PTB SNPs. A meta-analysis further confirmed the significant combined effect of LT, TNFRSF9, and IL20Ra in East Asian populations (*P* < 0.05).

**Conclusions:**

Levels of specific inflammatory proteins may play a crucial role in triggering an immune response to PTB. Altered levels of LT and TNFRSF9 have the potential to serve as predictive markers for PTB development, necessitating further clinical validation in real-world settings to ascertain the impact of these inflammatory proteins on PTB.

## 1 Introduction

The Mycobacterium tuberculosis (MTB) complex, which causes Pulmonary Tuberculosis (PTB) in humans and animals, is globally recognized as the second leading infectious cause of mortality, following novel coronavirus pneumonia (1). With the rapid advancement of molecular biology detection technology, enzyme-linked immunosorbent assay based on immunology technology exhibits excellent specificity. However, it is susceptible to individual immune status and other influencing factors, thereby necessitating high testing costs. GeneXpert MTB/RIF has gained widespread usage due to its advantages in terms of ease of operation, fast detection speed, and high sensitivity. Nevertheless, its diagnostic capability is limited to the period when Mycobacterium tuberculosis is excreted by patients with tuberculosis and cannot be employed during the non-excretory phase. Consequently, there exists an urgent need for a direct, rapid, and accurate method for early tuberculosis diagnosis (2). Several studies have identified certain inflammatory proteins as crucial players in PTB pathology, indicating a promising avenue for PTB diagnosis and targeted therapy (3, 4).

The eradication of PTB requires the concerted efforts of both cellular immunity and humoral immunity, with T cells contributing to cellular immunity and B cells playing a role in humoral immunity. MTB is a facultative intracellular bacterium that predominantly resides within macrophages (Mø). When aerosol droplets containing MTB are inhaled, MTB effectively recognizes alveolar Mø located on the lining of the alveoli. Mø exhibits limited ability to detect or respond reliably to MTB infection, resulting in a weakened inflammatory response. Ultimately, migration from the alveolar space to the lung interstitial occurs through host-dependent interleukin 1b (IL-1b) signaling and direct activity of other molecules, leading to a delay of adaptive immunity by 2 weeks (5). The involvement of B cells in anti-tuberculosis humoral immunity and their regulatory role in various host immune components contribute to the enhancement of cellular immune response against MTB. B cells not only produce antibodies but also secrete pro-inflammatory [such as interferon–gamma (IFN-γ) and tumor necrosis factor-alpha levels (TNF-α)] and anti-inflammatory cytokines (for instance, IL-4、IL-10). The role of B cells in promoting protective immunity and modulating host defense is substantial. The evidence regarding the contribution of B cells and antibodies to MTB clearance exhibits significant variability, necessitating further investigation into whether this disparity can be attributed to genetic factors within the study population (6). Historically, the majority of tuberculosis research has been focused on elucidating the mechanisms underlying T-cell-mediated immunity, while our understanding of the role played by B-cell and antibody-mediated immunity in tuberculosis remains incomplete. Given our current understanding of MTB’s immunological mechanism, it may be advantageous to explore molecular biological evidence of active infection from a cytokine-centric perspective.

The discovery of TNF originated from the observation that cancer patients occasionally exhibited spontaneous tumor regression following bacterial infection. Subsequent investigations have revealed its identity as a circulating factor induced by bacteria, possessing potent anti-tumor activity and referred to as TNF (7). Primarily implicated in apoptosis and inflammation, TNF receptors can also participate in other signal transduction pathways (8–10). The study of members within the TNF family will continue to be a prominent area of research. Studies have revealed that TNF-β, a soluble protein released by activated lymphocytes in response to antigen or mitogen stimulation, possesses the ability to inhibit tumor cells and virus-infected cell growth or lysis. This protein is also known as lymphotoxin-α (LT), which has been found to replace TNF-β in this paper (11). As a member of the TNF family, LT is widely acknowledged as a potent pro-inflammatory mediator. It effectively suppresses human granzyme B (GZMB) regulatory B cells (Bregs). Utilizing the geneHancer database and TFBSTools R package to identify transcription factors binding to LT and GZMB promoters, two upregulated transcription factors were discovered in GZMB+Bregs. Inhibiting LT in GZMB+Bregs can diminish the induction of GZMB+Bregs. GZMB+Bregs inhibit T cell proliferation and effector function by suppressing cytokine production and proliferation. Subsequent administration of LT restores effector T cell proliferation in a dose-dependent manner (12). The role of LT in the immune response of PTB involves the regulation of B cells and the promotion of T cell proliferation. Investigating whether LT can induce a shift from humoral immunity to cellular immunity is an innovative direction for future research on the diagnosis and treatment of PTB from an immunological perspective. The results of the experiments have demonstrated that LT possesses the capability to accurately detect MTB bovis infection upon stimulation with specific protein derivatives. The experiment exhibited a sensitivity of 0.9991, thereby confirming the diagnostic value of LT. Nevertheless, LT has received limited attention in tuberculosis research (13).

Given the impact of multiple inflammation-associated proteins on PTB infection and morbidity, we employed Mendelian randomization (MR) analysis, akin to a randomized controlled trial design, to elucidate the causal relationship between PTB and its associated inflammatory proteins through MR Validation in our study. The present study employed MR methods to evaluate the causal impact of inflammatory proteins’ genetic polymorphisms on PTB by utilizing single nucleotide polymorphisms (SNPs) associated with inflammatory proteins as instrumental variables (IVs).

## 2 Methods

### 2.1 Study design

The present study conducted a comprehensive evaluation of the association between 91 inflammatory proteins and PTB using a rigorous MR design. The conduction of a scientifically rigorous MR study necessitates the examination of the following three hypotheses: 1) genetic IVs exhibited a strong association with the exposure factors; 2) genetic IVs were found to be statistically uncorrelated with the outcomes and remained unaffected by potential confounding factors; 3) the results are influenced by IVs through their impact on exposure factors.

**Figure 1:**
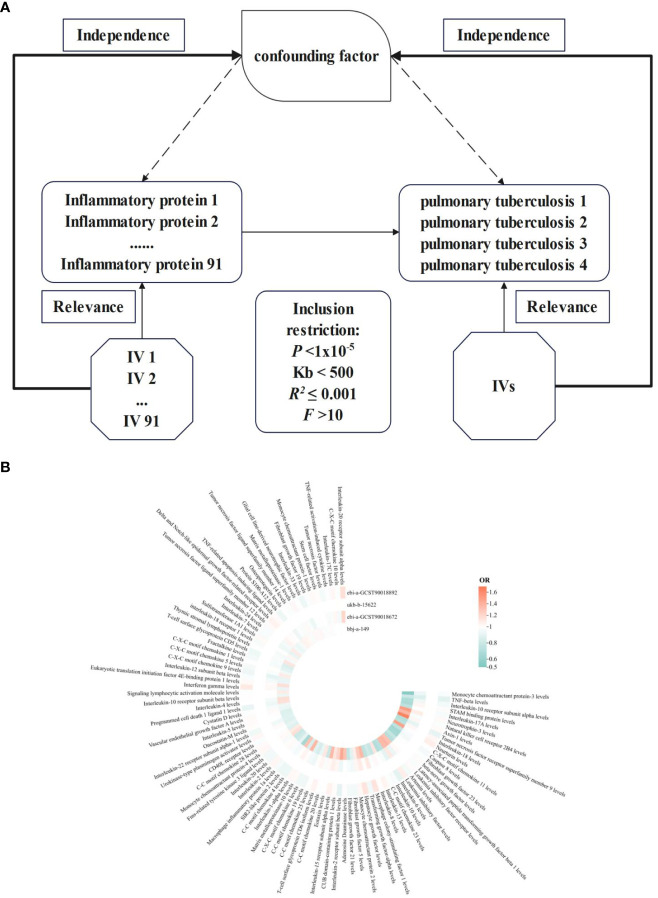
NIR flowcharts depicting 91 inflammatory proteins and their correlation significance across four PTB datasets. **(A)**, Schematic of MR analysis. Significant IVs for 91 inflammatory proteins and PTB were selected to explore causality. Three basic assumptions of MR analysis were also illustrated. In order to identify SNPs with a robust correlation between PTB and inflammatory proteins, it is commonly recommended to establish a genome-wide significance threshold of *P* < 1×10^-5^. When utilizing kb and *R^2^* to detect chain imbalance, it is typically advised to set Kb < 500 and *R^2^* ≤ 0.001, indicating the removal of SNPs within the range of 500 Kb and those with an *R^2^* value exceeding 0.001 as the most significant SNP. To assess tool efficacy using the *F* statistic, it is generally suggested to set *F* > 10. **(B)**, Circular heatmap plot of correlation significance of inflammatory proteins influence on PTB.

In a genome-wide protein quantitative trait locus (pQTL) study of 91 plasma proteins, MR Analysis was employed, utilizing a total of 91 inflammatory proteins and genetically significant SNPs associated with PTB. To mitigate potential sample bias, the inflammatory proteins and PTB genetic information utilized in this study were sourced from distinct Genome-wide association studies (GWAS) datasets (**Figure 1A**).

### 2.2 GWAS data for inflammatory proteins

The dataset utilized inflammatory protein data obtained from a European genome-wide pQTL study, which encompassed 14,824 participants at the Olink Target platform. The panel measured 92 inflammation-associated proteins using the Olink Target-96 Inflammation immunoassay panel. Proteomic data for each cohort were generated in Olink’s laboratory in Uppsala. During the project, Olink removed brain-derived neurotrophic factors from the inflammatory panel due to detection issues, so our study included a total of 91 proteins (14).

### 2.3 GEWAS data for PTB

The PTB pooled datasets were all obtained from the ieu open gewas project (https://gwas.mrcieu.ac.uk/). The GWAS IDs were ebi-a-GCST90018892, ukb-b-15622, bbj-a-149, and ebi-a-GCST90018672 respectively. SNPs were extracted from the VCF files shared by the analytic platforms. In the European Population PTB dataset with GWAS ID ebi-a-GCST90018892, the pooled data consisted of 895 PTB cases and 476,491 control cases, for a total of 24,189,689 SNPs. In the European Population PTB dataset with GWAS ID ukb-b-15622, the pooled data consisted of 2,277 PTB cases and 460,656 control cases, for a total of 9,851,867 SNPs. In the East Asian Population PTB dataset with GWAS ID bbj-a-149, the pooled data consisted of 549 PTB cases and 211,904 control cases, for a total of 8, 885, 805 SNPs. In the East Asian Population PTB dataset with GWAS ID ebi-a-GCST90018672, the pooled data consisted of 7800 PTB cases and 170,871 control cases, for a total of 12,454,677 SNPs.

### 2.4 Selection of IVs

The selection of IVs in this MR analysis was based on 3 basic assumptions. First, we set *P* < 1×10^-5^ as the genome-wide significance threshold to select SNPs strongly associated with PTB and inflammatory proteins. Secondly, to avoid linkage disequilibrium, a clustering procedure implemented in R software was used to identify independent variants. A threshold of *R^2^* < 0.001 at a distance of 500 kilobases was applied for assessing linkage disequilibrium. Finally, to quantitatively verify whether the selected SNPs were strong instruments, the proportion of variance in the exposure was calculated using the *R^2^* value for each SNPs, and instrument strength was estimated using the *F* statistic to avoid weak instrument bias. A threshold of *F* > 10 is usually recommended.

### 2.5 MR analysis

In this analysis, the causal relationship between inflammatory proteins and PTB was assessed primarily using the inverse variance weighting (IVW) method, which is the most efficient method with maximum statistical power. When the instrumental variables satisfy all three main assumptions, the IVW method provides a more accurate estimate of the causal effect of exposure and is considered the most efficient MR method. However, if some IVs do not meet the assumptions, the analysis may give inaccurate results. Therefore, we conducted the following sensitivity analysis:1) the hypothesis violation caused by the heterogeneity of the correlation between individual IV was examined through a *Q* test conducted on IVW and MR-Egger; 2) the MR-Egger intercept method was employed to estimate horizontal pleiotropy, ensuring the independent correlation between genetic variation and inflammatory proteins with PTB; 3) the reliability and stability of hypothesis testing are enhanced through the utilization of additional analysis techniques such as weighted median and weighted models; 4) the likelihood of observed associations for individual SNPs was assessed through the performance of individual SNP analyses and retention tests.

### 2.6 Colocalization analysis

Following the confirmation of a causal association between an inflammatory protein and PTB through MR Analysis, colocalization analysis identified significant signal sites to determine whether both phenotypes were influenced by the same causal variant in a specific region. This further strengthens the previous evidence linking these two phenotypes. The colocalization analysis consisted of four hypotheses. H_0_: There was no significant association between phenotype 1 (exposure) and phenotype 2 (outcome) with all SNPs in a genomic region. H_1_/H_2_: Either phenotype 1 or phenotype 2 showed significant association with SNP sites in a genomic region. H_3_: Both phenotypes 1 and 2 were significantly associated with SNP sites in a genomic region, but driven by different causal variation sites. H_4_: Both phenotypes 1 and 2 were significantly associated with SNP sites in a genomic region and driven by the same causal mutation site. The analysis provided posterior probabilities for each hypothesis test (H_0_, H_1_, H_2_, H_3_, and H_4_). If the posterior probability of shared causal variation is ≥0.75, it indicats strong evidence of colocalization between the SNP and both phenotypes.

### 2.7 Statistical analysis

All MR analyses were performed using the “TwoSampleMR” package in R (version 4.2.3). The odds ratio (*OR*) and the corresponding 95% confidence intervals (*CI*) were used to estimate the magnitude and direction of inflammatory protein effects. Meta-analysis was performed using Revman 5.4 software to collect the required data and create a database, statistical methods were used to analyze the heterogeneity using Heterogeneity, and also random and fixed effect models were used for the comparative analysis with a confidence interval of 95%. The colocalization analysis was conducted using the “coloc” package in R (version 4.2.3). *P* < 0.05 was considered statistically significant. The Heatmap plot of this sduty was created from https://www.chiplot.online/. The Venn and Volcano plot were generated from https://www.bioladder.cn/web/#/pro/index.

## 3 Results

### 3.1 Overall trends

The results of 91 inflammatory proteins from four databases were summarized and analyzed to determine their impact on the risk of PTB development. It was observed that the majority of inflammatory proteins have an *OR* value close to 1 in the development of PTB. Additionally, PTB exhibited divergent responses to 76 inflammatory proteins, while consistent effects were observed in 15 inflammatory proteins. Notably, levels of interleukin-10 receptor subunit alpha, neurotrophin-3, leukemia inhibitory factor receptor, interleukin-6, fibroblast growth factor 5 and 21, interleukin-15 receptor subunit alpha, T-cell surface glycoprotein CD6 isoform, C-C motif chemokine 19 and C-X-C motif chemokine 6 were found to be influential. Furthermore, the levels of interleukin-22 receptor subunit alpha-1 and eukaryotic translation initiation factor 4E-binding protein 1 as well as T-cell surface glycoprotein CD5 and sulfotransferase 1A1 also contributed to the development of PTB in conjunction with TNF-related activation-induced cytokine (**Figure 1B**). The integration effect and significance of this result should be further determined through meta-analysis.

### 3.2 Influence of 91 inflammatory proteins on PTB

**Figure 2:**
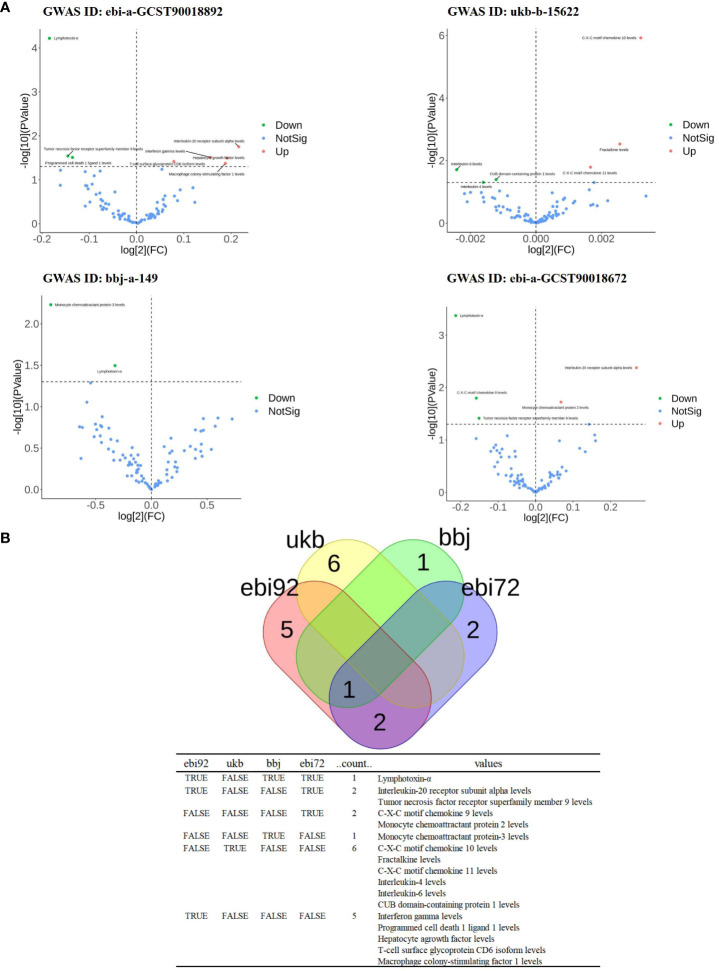
The significance and overlap of 91 inflammatory proteins across four PTB datasets. **(A)**, Volcano plot of correlation significance of inflammatory proteins influence on PTB in each database. **(B)**, Intersection of 4 databases in MR Analysis. The data set whose GWAS ID is ebi-a-GCST90018892 is abbreviated as ebi92. GWAS ID is the data set ukb-b-15622 is abbreviated as ukb. GWAS ID is the data set bbj-a-149 is abbreviated as bbj. The GWAS ID is ebi-a-GCST90018672 is abbreviated as ebi72.

**Figure 3:**
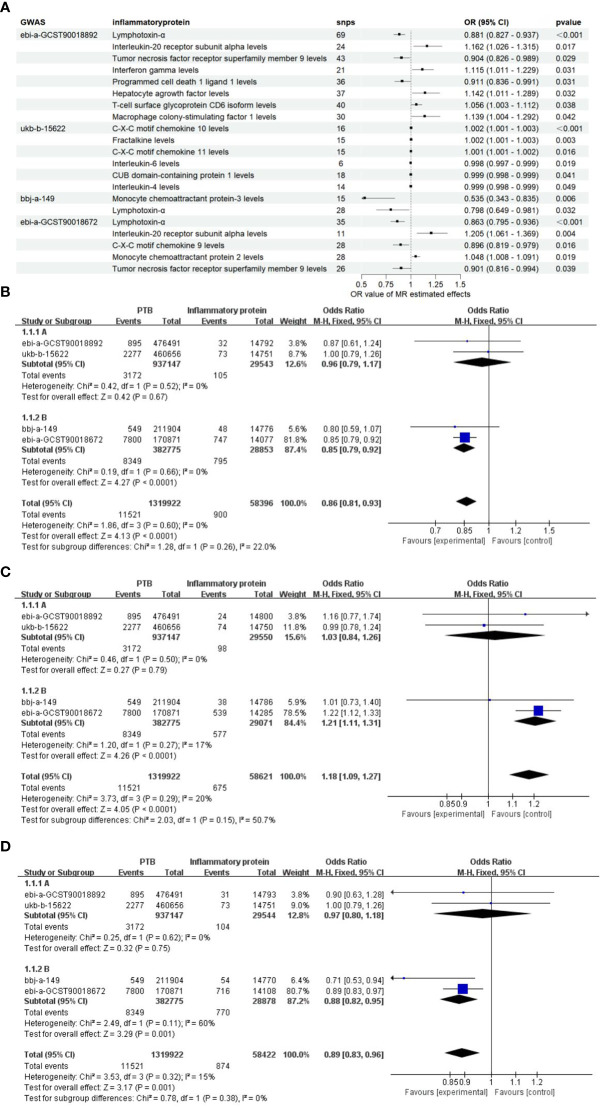
The *OR* values of inflammatory proteins exhibit a significant correlation. **(A)** The forest maps illustrate the importance of significant inflammatory proteins effects on PTB across four databases. **(B)**, Meta-analysis of TNF-β in four GWARS databases. **(C)**, Meta-analysis of TNFSF9 in four GWARS databases. **(D)**, Meta-analysis of IL20Ra in four GWARS databases.

Since the genome-wide significance threshold for selecting strongly correlated SNPs was *P* < 1×10^-5,^ there were 91 inflammatory proteins. The IVs variable for GWAS ID: ebi-a-GCST90018892 contains a total of 24,189, 689 SNPs, with a median of 37 SNPs. The IVs for GWAS ID: ukb-b-15622 contain a total of 9,851,867 SNPs, with a median of 13 SNPs. The IVs variable for GWAS ID: bbj-a-149 contain a total of 8,885,805 SNPs, with a median of 16 SNPs. The IVs for GWAS ID: ebi-a-GCST90018672 contains a total of 12,454,677 SNPs, with a median of 20 SNPs. All analyses used IVW as the primary analytical method, and there was no evidence of heterogeneity and no weak instruments. From the preliminary results of the study, 17 significantly correlated inflammatory proteins were screened from 4 databases (*P* < 0.05 for IVW) (**Figure 2A**). After the screening, LT, TNFSF9, and IL20Ra were identified with varying degrees of overlap (**Figure 2B**). The presence of LT demonstrated a protective effect against PTB in one European and two East Asian databases (*OR* < 1, *P* < 0.05). Similarly, TNFSF9 exhibited a protective effect against PTB in both a European and an East Asian population database (*OR* < 1, *P* < 0.05). Conversely, IL20Ra was identified as a risk factor for PTB in both a European and an East Asian population database (*OR* > 1, *P* < 0.05) (**Figure 3A**). The statistically significant correlations of three overlapping inflammatory proteins in the three MR Models, *Q* test, and sensitivity analysis are presented in **Supplementary Table 1**. All tool variables were found to be significant (*P* < 0.05).

### 3.3 Estimators of the combined effects

Considering the diverse causal relationships between various inflammatory proteins and PTB in different populations, we conducted a meta-subgroup analysis by pooling data from 4 databases. We investigated a total of three types of inflammatory proteins that exhibited statistically significant overlap, and performed heterogeneity tests as well as pooled effect size tests for the included studies. Additionally, we separately estimated the combined effects in European and East Asian populations based on population categories. The results of the meta-analysis demonstrated significant associations between LT, TNFRSF9, and IL20Ra with PTB in the East Asian population (*P* < 0.05). Furthermore, no heterogeneity was observed (*I^2^* < 50%, *P* > 0.05), and the final combined effect using the fixed-effect model was found to be significant (*P* < 0.05) (**Figures 3B-D**). Based on these findings, it can be concluded that LT, TNFRSF9, and IL20Ra exhibit significant associations with PTB in both the European population datasets and the datasets representing the East Asian population.

### 3.4 Colocalization analysis of LT

**Figure 4:**
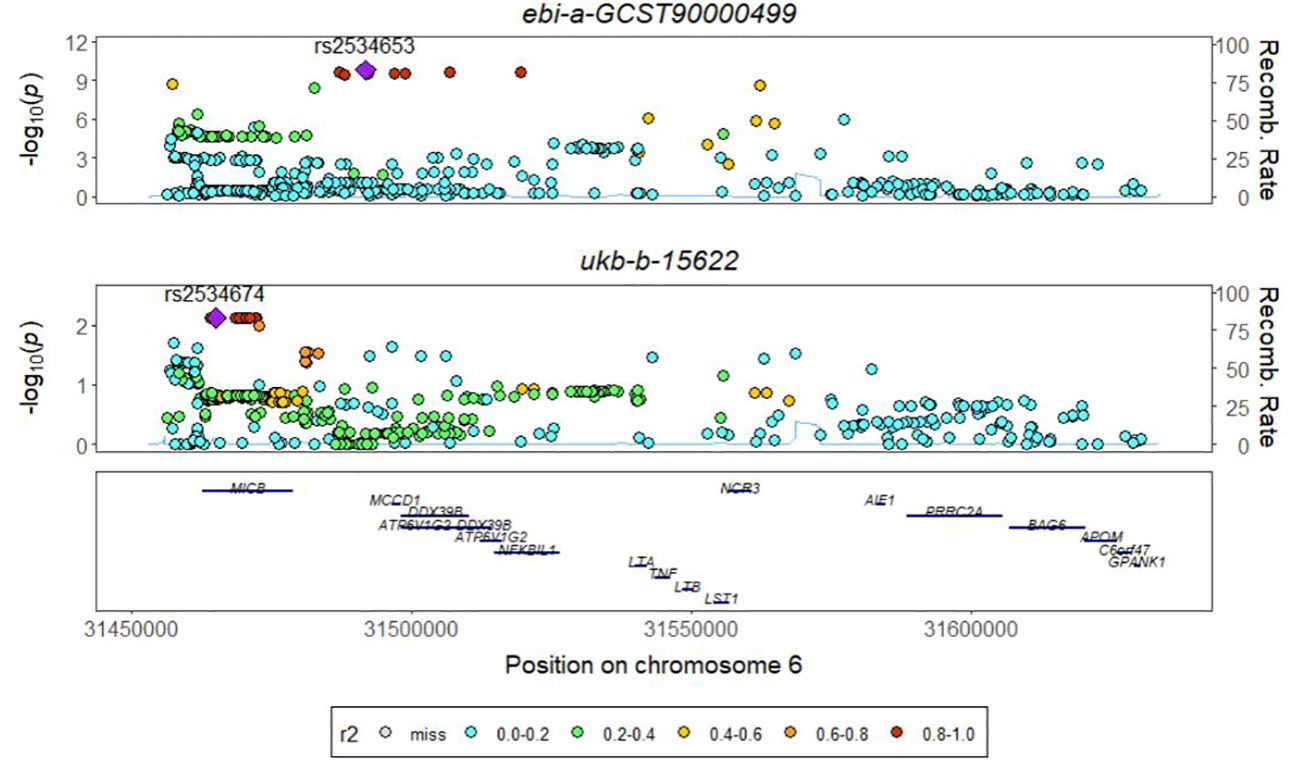
The colocalization results ofTNF-β and PTB.

The absence of a significant *P* value in the GWAS data for TNFRSF9 and IL20ra prompted us to conduct a co-localization analysis on LT, aiming to investigate whether there is shared underlying causal variation between LT and PTB within a specific genomic region. The results obtained demonstrate that the posterior probability of shared causal variation between LT and PTB is only 0.19%, which inadequately explains the causal relationship between LT and PTB (**Figure 4**, **Table 1**).

**Table 1:** The results of the co-localization analysis for LT were evaluated based on 4 distinct hypotheses.

Inflammatory protein	Chromosome and position	H_0_.abf*	H_1_.abf*	H_2_.abf*	H_3_.abf*	H_4_.abf*
LT	6: 31450757-31630757	8.47×10^-5^	9.96×10^-1^	1.79×10^-7^	2.11×10^-3^	1.90×10^-3^

*abf, Approximate Bayes Factors.

## 4 Discussion

In this study, we employed genetic variants as IVs to investigate the causal relationship between 91 inflammatory proteins as exposure variables and PTB in two European and two East Asian populations. Seventeen inflammatory proteins exhibited significant associations with PTB. The presence of LT and TNFSF9 may potentially confer a reduced susceptibility to PTB, whereas IL20Ra may increase the risk of developing PTB development, without observing any heterogeneity in these effects across European and East Asian populations. Furthermore, the posterior probability of shared causal variation between LT and PTB was only 0.19%, suggesting limited evidence for colocalization of the SNP with both phenotypes.

The present study underscored the causal role of TNF in determining susceptibility to PTB. Multiple studies have demonstrated that the diagnostic value of measuring TNF levels surpasses that of other factors (15). MR analysis offers novel insights into the causal relationship between inflammatory proteins and PTB pathogenesis. Research on the association between the TNF family and PTB is expanding. TNF plays a crucial role as a mediator against MTB, significantly influencing granulomatous structure, Bacillus colonization, and necrotic area maintenance. PTB is characterized by alterations in inflammatory markers, while systemic inflammation driven by MTB infection may exhibit diverse effects across different countries and ethnicities. The most relevant markers for distinguishing inflammatory characteristics among countries is TNF-α, which has garnered numerous research findings regarding its involvement in PTB pathogenesis. The pro-inflammatory response in subjects with PTB reached its peak at 2 months of treatment, as evidenced by an elevation in TNF-α levels, and subsequently exhibited down-regulation at 6 months of treatment (16). TNF-α is a prominent pro-inflammatory factor that triggers early inflammation in Mtb infection. Upon invasion of the body by MTB, TNF-α plays a crucial role in the innate immune response through its recruitment and activation of monocytes at the site of infection, thus enhancing their bactericidal activity. Furthermore, TNF-α serves as a key mediator of host immune responses and is secreted by activated Mø to combat PTB and restrict disease progression. It facilitates the formation and maintenance of granulomatous structures while promoting effector molecule production through macrophage activation when co-stimulated with IFN-γ (17, 18). The involvement of TNF-α in the regulation of inflammatory response during PTB is thus deemed highly significant. In clinical TB models, the virulent Peking strain exhibits an enhanced capacity to suppress lung protective immunity by inducing elevated levels of type I interferon, thereby leading to reduced TNF-α levels and diminished T cell activation when compared with strains from other lineages (19). Some studies of tuberculous pleurisy have reported elevated levels of TNF at the site of infection. Higher levels of TNF were observed in bronchoalveolar cells infected in the lungs of patients with unilateral TB compared to cells obtained from the unaffected side of the same patients. Additionally, higher levels of TNF were detected in peripheral blood mononuclear cells of patients newly diagnosed with TB compared to those with chronic refractory TB (20–22). Compared to Human Immunodeficiency Virus(HIV)–infected patients without central nervous system involvement, HIV-associated tuberculous meningitis patients exhibited elevated levels of T-helper factor (Th) 17, TNF-α, and LT (23). In the study conducted by Jing Wei et al., the Kyoto Genomic Encyclopedia and single gene enrichment analysis of peripheral blood mononuclear cells from the Asian population of Mtb-Ag stimulated and control samples revealed a significant enrichment of the TNF signaling pathway, which is closely associated with tuberculosis. Furthermore, it was observed that this pathway co-expressed with five pathways in the protein-protein interaction network, all of which exhibited upregulation under Mtb-Ag stimulation (24). Yukari C Manabe et al. conducted a randomized clinical trial in which 850 HIV patients from 10 African countries were evaluated for 26 plasma biomarkers using a combination of multiple immunoassays and enzyme-linked immunosorbent assays. The final model, constructed with six biomarkers including LT, effectively predicted the occurrence of PTB with a sensitivity of 0.90 and specificity of 0.71. These intuitive inflammatory biomarkers can be used to identify individuals at the highest risk for developing PTB (25). The association between LT and PTB is becoming increasingly evident, and it exerts a protective role during MTB infection. However, despite the ability of LT to induce the production of Th1 cytokines (such as interferon–amma, and IL-12) and promote pulmonary immune response, its independent effect appears to be insignificant. The absence of LT may compromise the control of chronic Mycobacterium tuberculosis infection, suggesting a potential involvement of LT in regulating chronic nodular MTB infection (26). These findings substantiate the efficacy of LT as a predictive factor for PTB. Moreover, among individuals co-infected with HIV and TB, the utilization of inflammatory biomarker signatures successfully identified those at the highest risk for developing PTB (23). These inflammatory biomarkers reflect numerous activations of innate immunity and increased Th1 responses, which are associated with the immune response to MTB. This characteristic holds promise as a stratification tool in the future and may benefit patients who require heightened monitoring and novel interventions. Based on our research findings, we intend to conduct the subsequent experiment on a large sample of the Asian population. We will establish an exposed group (with high levels of LT) and a non-exposed group (with low levels of LT) for cohort observation with the objective of validating whether elevated levels of LT can predict PTB occurrence across different ethnicities.

TNFRSF is a type of cytokine receptor consisting of superfamily proteins. TNFRSF9 is expressed on the surface of antigen-presenting cells, such as Mø and B cells, as well as various tumor cells. It plays diverse roles in autoimmune, infection, and inflammatory diseases by mediating complex immune responses (27, 28). However, their function extends beyond immune cells and also relates to the body’s pathological and physiological responses. This “double-edged sword” characteristic is typical among most members of the tumor necrosis factor family. Different studies have demonstrated that while TNF and its superfamily members were crucial for hematopoiesis, prevention of bacterial infection, immune surveillance, and tumor regression; their dysregulation also contributed to various diseases (29, 30). The regulatory network between TNFRSF9 and its ligand TNFSF9 is highly intricate, primarily due to the presence of bidirectional signaling that occurs between them. The interaction of TNFRSF9 and TNFSF9 on various cell types can initiate bidirectional signals, leading to a diverse range of immune responses involving both adaptive and innate immunity. However, the precise mechanisms underlying this complex regulatory network remain unclear (31). Therefore, TNFRSF9, which does not consistently exert a protective role in certain diseases, exhibits a distinctive functionality across various immune effector cell types due to its involvement in intricately orchestrated immune regulatory processes (32). The precise contribution of TNFRSF9 to human disease remains yet to be determined. Nonetheless, when combined with the findings from our study, these conclusions regarding TNF and TNFRSF9 offer potential for novel therapeutic target against TB.

The IL-20 family exerts a profound influence on the innate immune response of the host, promoting its activation and limiting the detrimental effects associated with viral and bacterial infections. Additionally, it facilitates tissue remodeling and repair processes while also playing a crucial role in restoring local epithelial homeostasis and preserving tissue integrity during episodes of inflammation and infection (33–36). The secretion and expression of inflammatory factors such as TNF-α and monocyte chemoattractant protein can be enhanced, thereby effectively regulating the occurrence of early inflammation. The data revealed a decrease in the production of these cytokines during active TB with diabetes and latent TB with diabetes, indicating their potential protective role against TB (37). In the context of tuberculosis, the mechanisms underlying the IL-20 family remain incompletely elucidated. The available findings suggest that the IL-20 subfamily of cytokines bears resemblance to IL-10 and exerts a regulatory role in modulating cytokine expression to suppress the inflammatory response during chronic inflammation (33). Building upon our discoveries, the exploration for novel ways to predict TB will go one step further.

This study should also acknowledge and strive to overcome certain limitations. The present study represents the first MR investigation aimed at assessing the causal association between PTB and a comprehensive panel of 91 inflammatory proteins. The susceptibility to confounding is relatively lower in MR Designs compared to other observational studies; however, there are still limitations. Firstly, the data we investigated originated from four large-scale GWAS, and due to the absence of specific demographic information and clinical records of the study participants, a more detailed analysis was not feasible. Second, the genetic association between exposure and outcome GWAS may exhibit population-specific variations and differential influences based on race. Our findings were derived from European and East Asian populations, which may differ from other populations; however, the fundamental biological link between systemic inflammatory proteins and PTB should remain consistent across populations. Therefore, caution should be exercised when extrapolating these conclusions to other racial groups. Third, the analysis was restricted to alterations in blood tissue, thus excluding the elucidation of discrepancies in other tissues among PTB patients. Fourth, the impact on PTB risk was the sole focus of our testing, without considering their influence on the progression of PTB disease. The investigation of the association between all systemic inflammatory regulators and disease progression, would be invaluable for comprehending PTB.

## 5 Conclusions

Taken collectively, the findings of the MR study suggest that levels of specific inflammatory proteins may play a pivotal role in triggering an immune response to PTB, thereby enabling the prediction of PTB development risk. Changes in levels of LT and TNFRSF9 may potentially serve as predictive markers for the development of PTB. Additionally, the effects of LT, TNFRSF9, and IL20Ra on PTB vary among different ethnic groups. However, further clinical validation in real-world settings is necessary to determine the impact of these inflammatory proteins on PTB.

## Data availability statement

The original contributions presented in the study are included in the article/**Supplementary Material**. Further inquiries can be directed to the corresponding authors.

## Ethics statement

This study was conducted using publicly available data and did not require ethical approval.

## Author contributions

WM: Data curation, Formal analysis, Investigation, Software, Validation, Visualization, Writing – original draft. ZC: Conceptualization, Formal analysis, Funding acquisition, Methodology, Project administration, Resources, Software, Supervision, Writing – review & editing. JZ: Visualization, Writing – original draft. XX: Data curation, Formal analysis, Investigation, Validation, Visualization, Writing – original draft. MH: Validation, Writing – review & editing. JL: Data curation, Funding acquisition, Methodology, Project administration, Validation, Writing – review & editing.

## References

[1] Bagcchi S. WHO's global tuberculosis report 2022. Lancet Microbe. (2023) 4:e20. doi: 10.1016/S2666-5247(22)00359-7 36521512

[2] Wang F Hou HY Wu SJ Zhu Q Huang M Yin B. Using the TBAg/PHA ratio in the T-SPOT^®^.*TB* assay to distinguish TB disease from LTBI in an endemic area. Int J Tuberc Lung Dis. (2016) 20(4):487–93. doi: 10.5588/ijtld.15.0756 26970158

[3] Jia HY Dong J Zhang ZD PAN LP. Progress and clinical application of immunological detection technology- for Ms-cobacterium tuberculosis infection. Chin J Antituberculosis. (2022) 44:720–6. doi: 10.19982/j.issn.1000-6621.20220103

[4] Li H Ren W Liang Q Zhang X Li Q Shang Y. A novel chemokine biomarker to distinguish active tuberculosis from latent tuberculosis: a cohort study. QJM. (2023) 116:1002–9. doi: 10.1093/qjmed/hcad214 PMC10753411 37740371

[5] Li N Song YJ Chu YF. Advances in immune escape mechanisms of Mycobacterium tuberculosis (in Chinese). Chin Sci Bull. (2023) 69(4):531–41. doi: 10.1360/TB-2023-0818

[6] Singh K Kumar R Umam F Kapoor P Sinha S Aggarwal A. Distinct and shared B cell responses of tuberculosis patients and their household contacts. PloS One. (2022) 17:e0276610. doi: 10.1371/journal.pone.0276610 36282846 PMC9595562

[7] Pinton P Braicu C Nougayrede JP Laffitte J Taranu I Oswald IP. Deoxynivalenol impairs porcine intestinal barrier function and decreases the protein expression of claudin-4 through a mitogen-activated protein kinase-dependent mechanism. J Nutr. (2010) 140:1956–62. doi: 10.3945/jn.110.123919 20861219

[8] Peterson LW Artis. Intestinalepithelial cells: Regulators of barrier function and immune homeostasis. Nat Rev Immunol. (2014) 14:141–53. doi: 10.1038/nri3608 24566914

[9] Jang DI Lee AH Shin HY. The role of tumor necrosis factor alpha (TNF-α) in autoimmune disease and current TNF-α Inhibitors in therapeutics. Int J Mol Sci. (2021) 22:2719. doi: 10.3390/ijms22052719 33800290 PMC7962638

[10] SABIO G. DAVIS R J.TNF and MAP kinase signalling pathways. Seminarsin Immunol. (2014) 26:237–45. doi: 10.1016/j.smim.2014.02.009 PMC4099309 24647229

[11] Cheng Y Liu Y Xu D Zhang D Yang Y Miao Y. An engineered TNFR1-selective human lymphotoxin-alpha mutant delivered by an oncolytic adenovirus for tumor immunotherapy. Biochim Biophys Acta Mol Basis Dis. (2024) 870(5):167122. doi: 10.1016/j.bbadis.2024.167122 38492783

[12] Sailliet N Mai HL Dupuy A Tilly G Fourgeux C Braud M. Human granzyme B regulatory B cells prevent effector CD4+CD25- T cell proliferation through a mechanism dependent from lymphotoxin alpha. Front Immunol. (2023) 14:1183714. doi: 10.3389/fimmu.2023.1183714 37588598 PMC10425555

[13] Fang L Lin W Jia H Gao X Sui X Guo X. Potential diagnostic value of the peripheral blood mononuclear cell transcriptome from cattle with bovine tuberculosis. Front Vet Sci. (2020) 7:295. doi: 10.3389/fvets.2020.00295 32528988 PMC7266948

[14] Zhao JH Stacey D Eriksson N Macdonald-Dunlop E Hedman ÅK Kalnapenkis A. Genetics of circulating inflammatory proteins identifies drivers of immune-mediated disease risk and therapeutic targets published correction appears in Nat Immunol. Nat Immunol. (2023) 24:1540–51. doi: 10.1038/s41590-023-01635-6 PMC10457199 37563310

[15] da Cunha Lisboa V Ribeiro-Alves M da Silva Corrêa R Ramos Lopes I Mafort TT. Predominance of Th1 immune response in pleural effusion of patients with tuberculosis among other exudative etiologies. J Clin Microbiol. (2019) 58:e00927–19. doi: 10.1128/JCM.00927-19 PMC6935897 31619524

[16] Okeke CO Amilo GI Manafa PO Ibeh NC. Inflammation-mediated changes in haemostatic variables of pulmonary tuberculosis patients during treatment. Tuberc Edinb. (2023) 138:102285. doi: 10.1016/j.tube.2022.102285 36436460

[17] Kathamuthu GR Sridhar R Baskaran D Babu S. Low body mass index has minimal impact on plasma levels of cytokines and chemokines in tuberculous lymphadenitis. J Clin Tuberc Other Mycobact Dis. (2020) 20:100163. doi: 10.1016/j.jctube.2020.100163 32420460 PMC7218292

[18] Fernández Do Porto DA Jurado JO Pasquinelli V Alvarez IB Aspera RH Musella RM. CD137 differentially regulates innate and adaptive immunity against Mycobacterium tuberculosis. Immunol Cell Biol. (2012) 90:449–56. doi: 10.1038/icb.2011.63 PMC3330265 21747409

[19] Mourik BC de Steenwinkel JEM de Knegt GJ Huizinga R Verbon A Ottenhoff THM. Mycobacterium tuberculosis clinical isolates of the Beijing and East-African Indian lineage induce fundamentally different host responses in mice compared to H37Rv. Sci Rep. (2019) 9:19922. doi: 10.1038/s41598-019-56300-6 31882653 PMC6934500

[20] Barnes PF Fong SJ Brennan PJ Twomey PE Mazumder A Modlin RL. Local production of tumor necrosis factor and IFN-gamma in tuberculous pleuritis. J Immunol. (1990) 145:149–54. doi: 10.4049/jimmunol.145.1.149 2113553

[21] Law K Weiden M Harkin T Tchou-Wong K Chi C Rom WN. Increased release of interleukin-1 beta, interleukin-6, and tumor necrosis factor-alpha by bronchoalveolar cells lavaged from involved sites in pulmonary tuberculosis. Am J Respir Crit Care Med. (1996) 153:799–804. doi: 10.1164/ajrccm.153.2.8564135 8564135

[22] Takashima T Ueta C Tsuyuguchi I Kishimoto S. Production of tumor necrosis factor alpha by monocytes from patients with pulmonary tuberculosis. Infect Immun. (1990) 58:3286–92. doi: 10.1128/iai.58.10.3286-3292.1990 PMC313651 2205576

[23] Xu L Xu Y Zheng Y Peng X Yang Z Cao Q. Differences in cytokine and chemokine profiles in cerebrospinal fluid caused by the etiology of cryptococcal meningitis and tuberculous meningitis in HIV patients. Clin Exp Immunol. (2021) 206:82–90. doi: 10.1111/cei.13644 34287847 PMC8446401

[24] Wei J Guo F Song Y Xu K Lin F Li K. Transcriptional analysis of human peripheral blood mononuclear cells stimulated by Mycobacterium tuberculosis antigen. Front Cell Infect Microbiol. (2023) 13:1255905. doi: 10.3389/fcimb.2023.1255905 37818041 PMC10561294

[25] Manabe YC Andrade BB Gupte N Leong S Kintali M Matoga M. A parsimonious host inflammatory biomarker signature predicts incident tuberculosis and mortality in advanced human immunodeficiency virus. Clin Infect Dis. (2020) 71:2645–54. doi: 10.1093/cid/ciz1147 PMC7744990 31761933

[26] Allie N Keeton R Court N. Limited role for lymphotoxin α in the host immune response to Mycobacterium tuberculosis. J Immunol. (2010) 185:4292–301. doi: 10.4049/jimmunol.1000650 20817877

[27] Melero I Murillo O Dubrot J Hervás-Stubbs S Perez-Gracia JL. Multi-layered action mechanisms of CD137 (4-1BB)-targeted immunotherapies. Trends Pharmacol Sci. (2008) 29(8):383–90. doi: 10.1016/j.tips.2008.05.005 18599129

[28] Shuh M Bohorquez H Loss GE Jr Cohen AJ. Tumor necrosis factor-α: life and death of hepatocytes during liver ischemia/reperfusion injury. Ochsner J. (2013) 13:119–30. PMC3603175 23531747

[29] Dostert C Grusdat M Letellier E Brenner D. The TNF family of ligands and receptors: communication modules in the immune system and beyond. Physiol Rev. (2019) 99:115–60. doi: 10.1152/physrev.00045.2017 30354964

[30] Sonar S Lal G. Role of tumor necrosis factor superfamily in neuroinflammation and autoimmunity. Front Immunol. (2015) 6:364. doi: 10.3389/fimmu.2015.00364 26257732 PMC4507150

[31] Nedoszytko B Szczerkowska-Dobosz A Stawczyk-Macieja M Owczarczyk-Saczonek A Reich A. Pathogenesis of psoriasis in the "omic" era. Part II. Genetic, genomic and epigenetic changes in psoriasis. Postepy Dermatol Alergol. (2020) 37:283–98. doi: 10.5114/ada.2020.96243 PMC7394158 32774210

[32] He Y Vlaming M van Meerten T Bremer E. The implementation of TNFRSF co-stimulatory domains in CAR-T cells for optimal functional activity. Cancers (Basel). (2022) 14:299. doi: 10.3390/cancers14020299 35053463 PMC8773791

[33] Kumar NP Moideen K Banurekha VV Nair D Babu S. Modulation of Th1/Tc1 and Th17/Tc17 responses in pulmonary tuberculosis by IL-20 subfamily of cytokines. Cytokine. (2018) 108:190–6. doi: 10.1016/j.cyto.2018.04.005 PMC5962435 29684756

[34] Fonseca-Camarillo G Furuzawa-Carballeda J Llorente L Yamamoto-Furusho JK. IL-10– and IL-20–expressing epithelial and inflammatory cells are increased in patients with ulcerative colitis. J Clin Immunol. (2013) 33:640–8. doi: 10.1007/s10875-012-9843-4 23207823

[35] Ouyang W Rutz S Crellin NK Valdez PA Hymowitz SG. Regulation and functions of the IL-10 family of cytokines in inflammation and disease. Annu Rev Immunol. (2011) 29:71–109. doi: 10.1146/annurev-immunol-031210-101312 21166540

[36] Cui XF Cui XG Leng N. Overexpression of interleukin 20 receptor subunit beta (IL20RB) correlates with cell proliferation, invasion and migration enhancement and poor progno sis in papillary renal cell carcinoma. J Toxicol Pathol. (2019) 32:245–51. doi: 10.1293/tox.2019-0017 PMC6831501 31719751

[37] Kumar NP Banurekha VV Nair D Kumaran P Dolla CK Babu S. Type 2 diabetes - Tuberculosis co-morbidity is associated with diminished circulating levels of IL-20 subfamily of cytokines. Tuberc (Edinb). (2015) 95:707–12. doi: 10.1016/j.tube.2015.06 PMC4666794 26354610

