# Supplementary material for: The predictive value of TNF family for pulmonary tuberculosis: a pooled causal effect analysis of multiple datasets
Source: Front Immunol. 2024 May 21;15:1398403. doi: 10.3389/fimmu.2024.1398403 (PMC11148272; doi:10.3389/fimmu.2024.1398403)
Supplement: Supplementary file 1 [file Table_1.docx]

**SUPPLEMENTARY TABLE 1.** Three MR models estimate the causal relationships between 3 statistically significant inflammatory proteins and the risk of PTB and tests for heterogeneity and horizontal pleiotropy. WM, weighted median; IVW, inverse variance weighted.

| Inflammatory proteins | GWAS ID | Methods | SNP N) | *OR*  (95% CI) | *P* | Heterogeneity | *P* | Pleiotropy | *P* |
| --- | --- | --- | --- | --- | --- | --- | --- | --- | --- |
|  |  |  |  |  |  | Q value (I2) |  | Intercept |  |
| TNF-β | ebi-a-GCST90018892 | IVW | 69 | 0.88 (0.83-0.94) | < 0.001 | 127.785 | < 0.001 | 0.005 | 0.577 |
|  |  | MR Egger | 69 | 0.86 (0.78-0.95) | 0.004 | 127.190 | < 0.001 |  |  |
|  |  | WM | 69 | 0.90 (0.84-0.97) | 0.004 |  |  |  |  |
|  | bbj-a-149 | IVW | 28 | 0.80 (0.65-0.98) | 0.032 | 32.720 | 0.206 | -0.030 | 0.339 |
|  |  | MR Egger | 28 | 0.90 (0.65-1.25) | 0.543 | 31.569 | 0.208 |  |  |
|  |  | WM | 28 | 0.86 (0.67-1.11) | 0.239 |  |  |  |  |
|  | ebi-a-GCST90018672 | IVW | 35 | 0.86 (0.79-0.94) | < 0.001 | 94.331 | < 0.001 | 0.005 | 0.694 |
|  |  | MR Egger | 35 | 0.85 (0.74-0.96) | 0.016 | 93.883 | < 0.001 |  |  |
|  |  | WM | 35 | 0.90 (0.84-0.96) | 0.003 |  |  |  |  |
| TNFRSF9 | ebi-a-GCST90018892 | IVW | 43 | 0.90 (0.83-0.99) | 0.029 | 43.095 | 0.424 | -0.019 | 0.157 |
|  |  | MR Egger | 43 | 1.08 (0.84-1.39) | 0.567 | 41.019 | 0.470 |  |  |
|  |  | WM | 43 | 0.95 (0.83-1.09) | 0.489 |  |  |  |  |
|  | ebi-a-GCST90018672 | IVW | 26 | 0.90 (0.82-0.99) | 0.039 | 27.089 | 0.351 | -0.013 | 0.403 |
|  |  | MR Egger | 26 | 1.02 (0.76-1.37) | 0.909 | 26.295 | 0.338 |  |  |
|  |  | WM | 26 | 0.93 (0.81-1.07) | 0.323 |  |  |  |  |
| IL20Ra | ebi-a-GCST90018892 | IVW | 24 | 1.16 (1.03-1.32) | 0.018 | 25.056 | 0.347 | -0.011 | 0.538 |
|  |  | MR Egger | 24 | 1.29 (0.91-1.81) | 0.165 | 24.619 | 0.315 |  |  |
|  |  | WM | 24 | 1.25 (1.06-1.47) | 0.007 |  |  |  |  |
|  | ebi-a-GCST90018672 | IVW | 11 | 1.21 (1.06-1.37) | 0.004 | 8.240 | 0.605 | -0.017 | 0.364 |
|  |  | MR Egger | 11 | 1.43 (0.98-2.09) | 0.094 | 7.328 | 0.603 |  |  |
|  |  | WM | 11 | 1.30 (1.09-1.54) | 0.003 |  |  |  |  |

*The values below 0.001 are represented as < 0.001.
